# Combating ageing beyond the cell: Emerging roles of extracellular proteostasis

**DOI:** 10.1111/febs.70647

**Published:** 2026-07-12

**Authors:** Siddharth R. Venkatesh, Ivan Gallotta, Bolanle F. Olabiyi, Yoon Hee Choi, Sarah Inglesfield, Janet R. Kumita, Collin Y. Ewald, Della C. David

**Affiliations:** ^1^ Signalling Programme Babraham Institute Cambridge UK; ^2^ Bioinformatics Facility Babraham Institute Cambridge UK; ^3^ Department of Pharmacology University of Cambridge UK; ^4^ Laboratory of Extracellular Matrix Regeneration, Institute of Translational Medicine, Department of Health Sciences and Technology ETH Zürich Schwerzenbach Switzerland; ^5^ Present address: University College London UK

**Keywords:** ageing, extracellular chaperones, extracellular matrix, extracellular proteases, extracellular proteostasis network, immunity, protein aggregation

## Abstract

Proteostasis, the maintenance of a healthy proteome, is a fundamental pillar of cellular and organismal health that declines with age. While the intracellular proteostasis network (PN) is well‐characterised, proteostasis mechanisms acting in the extracellular space remain understudied. Yet, these mechanisms face unique challenges and are critical for ensuring functional systemic signalling, immune surveillance and structural integrity. In contrast to the cytosol, extracellular environments lack ATP‐activated chaperones and a comprehensive ubiquitin‐proteasome system and instead rely on specialised secreted chaperones, extracellular proteases and receptor‐mediated clearance mechanisms. This review examines the emerging landscape of the extracellular proteostasis network (exPN) and its challenges with age. We discuss how age‐related remodelling of the extracellular proteome, shifts in extracellular physicochemical properties and disrupted fluid dynamics collectively create a permissive environment for protein misfolding and aggregation. We evaluate current experimental models of extracellular protein damage and examine how exPN factors target specific stages of the aggregation process to cooperatively safeguard extracellular proteome integrity. Analysis of recent human proteomic data spanning the life course uncovers an unexpected upregulation of exPN components with age. We further explore the role of extracellular proteostasis in inflammageing, a defining hallmark of ageing. Finally, we highlight strategies that bolster extracellular proteostasis as a promising frontier for extending healthspan, limiting age‐associated protein aggregation and restoring extracellular matrix homeostasis. By adopting an ageing‐centred perspective, we move beyond the disease context to present a holistic overview of extracellular proteostasis in organismal health, thereby positioning the exPN as a critical yet under‐exploited target for biomedical intervention.

## Graphical abstract

Ageing challenges the body's ability to maintain a stable and functional proteome, leading to protein damage and aggregation both inside and outside cells. This review focuses on the less well understood mechanisms of extracellular protein quality control and how they become disrupted in ageing, particularly in neurodegenerative diseases. We highlight interactions with the immune system and growing evidence that boosting extracellular protein maintenance can help support healthier ageing.

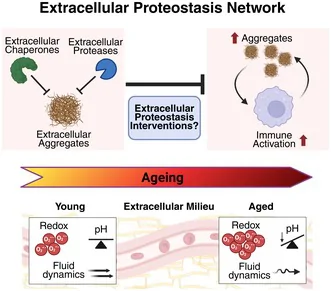

**Abbreviations**
AD: Alzheimer's disease
AGEs: Advanced glycation end products
AMPs: Antimicrobial peptides
ASC: Apoptosis‐associated speck‐like protein containing a caspase recruitment domain
CSF: Cerebrospinal fluid
EC: Extracellular chaperones
ECM: Extracellular matrix
ER: Endoplasmic reticulum
exPN: Extracellular proteostasis network
hIAPP: Human islet amyloid polypeptide
HOCl: Hypochlorous acid
MMP: Matrix metalloproteinase
NS: Neuroserpin
PQC: Protein quality control
RAGE: Receptor for advanced glycation end product
SAP: Serum amyloid P component
SASP: Senescence‐associated secretory phenotype
sHSPs: Small heat shock proteins
SPARC: Secreted protein, acidic, rich in cysteine
ThT: Thioflavin‐T
TIMP2: Tissue inhibitor of metalloproteinases 2
TLR: Toll‐like receptor
tPA: Tissue‐type plasminogen activator
TREM2: Triggering receptor expressed on myeloid cells 2
TTR: Transthyretin

## Introduction

The process of ageing is characterised by the gradual decline in physiological function across tissues and organs, and increased susceptibility to a range of diseases, including cardiovascular disorders, neurodegeneration, cancer and fibrotic conditions [1]. Loss of proteostasis is a primary hallmark of ageing and has emerged as a likely driver of age‐associated dysfunction at the cellular and organismal levels. Proteostasis encompasses the mechanisms that regulate protein synthesis, folding, trafficking, secretion and degradation. While intracellular proteostasis has been extensively studied, recent advances highlight the emerging role of extracellular proteostasis in preventing the accumulation of protein aggregates in the extracellular space.

Unlike the intracellular space, the extracellular milieu lacks ATP‐dependent chaperones and proteasomes, relying instead on secreted chaperones (e.g. clusterin and α_2_‐macroglobulin), extracellular proteases, extracellular matrix (ECM) turnover and receptor‐mediated clearance pathways to maintain protein solubility and function [2, 3, 4, 5, 6, 7, 8]. These mechanisms become less effective with age, leading to progressive extracellular protein misfolding, ECM stiffening, crosslinking and aggregation.

Because extracellular proteins mediate cell–cell signalling, immune surveillance, metabolic coordination and tissue architecture, their dysregulation can drive systemic ageing phenotypes, inflammageing and functional decline across organ systems. Therefore, understanding extracellular proteostasis is essential for explaining organismal ageing, identifying therapeutic targets and developing interventions that restore tissue homeostasis.

This review explores extracellular proteostasis in the context of ageing by outlining age‐related challenges for the extracellular proteome, surveying experimental models, describing the mechanisms that limit protein aggregation and assessing deregulation of the extracellular proteostasis network (exPN) in ageing and disease, before discussing its interplay with the immune system, and highlighting strategies to strengthen extracellular proteostasis for healthy ageing.

## Age‐related changes to the extracellular proteome

**Fig. 1:**
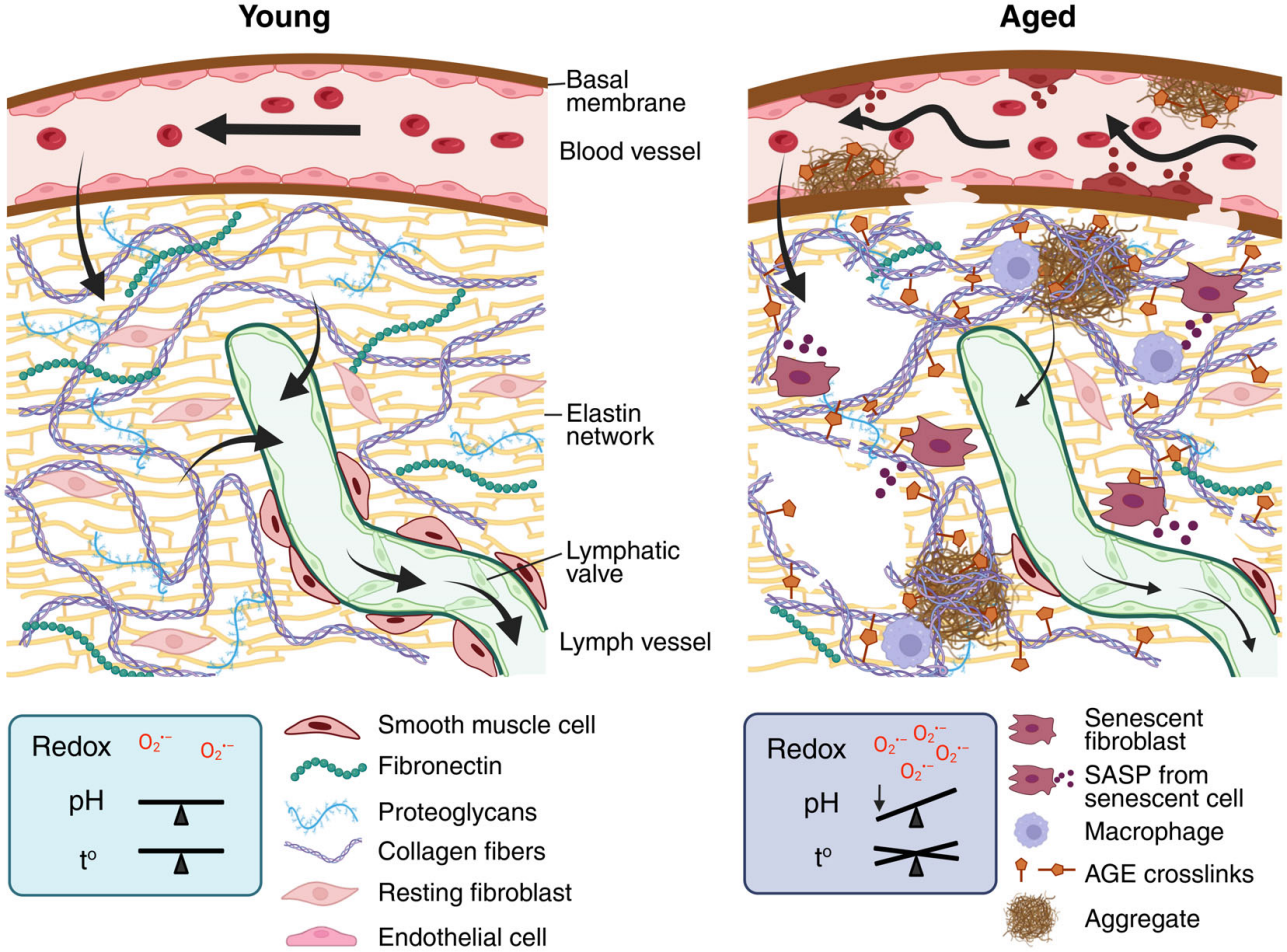
Age‐related challenges to extracellular proteostasis. Diagram depicting age‐dependent changes across extracellular compartments, including vasculature, interstitial space and lymphatic system. Ageing disrupts ECM turnover at the basal membranes and within the interstitial space, leading to areas of weaker ECM alongside accumulations of stiff, AGE‐crosslinked ECM domains. Physicochemical changes and AGE modifications increase protein damage, promoting deposition of protein aggregates in both vasculature and interstitial spaces, triggering macrophage activation. Senescent fibroblasts and endothelial cells contribute to a pro‐inflammatory milieu by releasing SASP factors. Concurrently, the loss of lymphatic muscle cells impairs interstitial fluid drainage and reduces the clearance of damaged proteins, further compromising extracellular proteostasis. AGE, advanced glycation end products; ECM, extracellular matrix; SASP, senescence‐associated secretory phenotype. Created in BioRender. David, D. (2026) https://BioRender.com/n2d0485.

Changes in the extracellular proteome are key features of systemic ageing across the organism. The plasma proteome, one of the most extensively characterised proteomes, exhibits distinct age‐related changes, which have enabled the development of proteomic clocks predicting biological ageing rates [9]. Notably, ECM proteins and secreted ECM‐remodelling factors alone are sufficient to construct a biological ageing clock with predictive power comparable to full plasma proteomic ageing clocks [10]. Parabiosis experiments have causally implicated circulating factors such as β2‐microglobulin [11] and CCL11 chemokine [12] as drivers of ageing. The age‐dependent shifts in plasma composition are closely linked to the differential ageing of specific organs producing these proteins [13, 14, 15, 16]. Age‐related changes in the extracellular proteome are partly driven by increased secretion of pro‐inflammatory mediators from senescent cells, forming the senescence‐associated secretory phenotype (SASP) that becomes more pronounced as these cells accumulate in ageing tissues (Fig. 1). The early ageing of the vasculature and immune system emerges as potentially key contributors to the pro‐ageing secretory signal driving systemic ageing [15].

With age, the ECM undergoes widespread compositional changes alongside a breakdown in the precise balance between its synthesis and degradation [8, 17] (Fig. 1). This is especially evident in altered collagen dynamics, which are central to maintaining ECM structural integrity [18]. While reduced collagen deposition is linked to human skin ageing [19], excessive collagen accumulation with age is associated with basal membrane thickening and stiffness across many organs, especially in the kidney, liver and cardiovascular system [20, 21]. Age‐related changes in the ECM composition and thickness fundamentally alters baseline tissue mechanics and stiffness, initiating a pathological mechanobiological feedback loop where aberrant integrin activation and altered mechanical compliance compromise cellular homeostasis [22]. These ECM‐related homeostasis changes are correlated with tissue decline, as observed in many age‐related diseases, including fibrosis, cancer and osteoarthritis [23].

Alongside shifts in the extracellular proteome, ageing destabilises the physicochemical environment outside cells, creating conditions that favour protein misfolding and aggregation. Aggregation often results in a loss of native function and conformational changes leading to insoluble protein deposits, including amyloid aggregates where proteins undergo compact rearrangement into stacks of cross‐β sheet fibrils [24]. Importantly, protein aggregation is not merely a consequence of ageing but can drive cellular dysfunction through proteotoxic stress, inflammatory activation and disruption of extracellular signalling networks [25, 26, 27, 28, 29]. Abundant extracellular deposition of protein aggregates in select tissues or systemically is a major pathological hallmark in devastating diseases, such as neurodegenerative diseases and systemic amyloidosis [30, 31]. These include aggregation of Aβ in Alzheimer's, PrP^sc^ in Prion diseases, islet amyloid polypeptide (IAPP) in type‐2 diabetes and immunoglobulin light chain in systemic amyloidosis. More widespread subclinical extracellular aggregate deposition and increased secretion of amyloidogenic proteins is now recognised as a hallmark of ageing tissues [15, 28, 32, 33, 34]. Studies of protein aggregation in the plasma suggest increased levels of circulating aggregates with age [35, 36]. Arteries are both a major source and deposition site for protein aggregates. Notably, arteries show prominent staining for Serum amyloid P component (SAP), which binds to aggregates with high affinity [15], and amyloidogenic medin peptides line the aortas of most individuals over 50 years of age [37, 38] (Fig. 1). While fibrin polymerisation into insoluble fibrils is a normal physiological protein‐aggregation process essential for blood clotting, this process can become dysregulated, leading to pathological amyloidogenic fibrin clots [39], associated with vascular ageing and disease. In addition, growing evidence suggests that cells under proteotoxic stress can extrude intracellular aggregates into the extracellular space. This mechanism may contribute to pathology propagation in neurodegeneration [40], and studies in *Caenorhabditis elegans* (*C. elegans*) show that proteostressed neurons also release aggregated proteins into the body cavity, suggesting that extracellular disposal of poorly degradable protein material is evolutionarily conserved [41].

Several studies have demonstrated that extracellular protein aggregation acts as a primary pathological driver by triggering sterile inflammation [28, 42]. Aggregates may cause further damage by sequestering functional proteins [43, 44], cross‐seeding other aggregates [45, 46, 47, 48] and interfering with signalling cascades [29, 49, 50, 51, 52].

## Physicochemical changes contributing to proteostasis imbalance

Compared with intracellular conditions, the physicochemical properties and age‐related changes in the extracellular fluids, including oxidation, sugar adducts, pH and temperature changes, pose an additional challenge for maintaining protein stability (Fig. 1). The extracellular environment is in a highly oxidising redox state and under physiological conditions, hydrogen peroxide concentrations in blood plasma are approximately 100 times higher than those found within cells [53]. Despite some dedicated extracellular redox components, such as GPx‐3/GPx‐P, EC‐SOD and Peroxiredoxin‐4, extracellular fluids have limited capacity to avoid oxidative damage [54]. Moreover, the plasma steadily becomes more oxidised with age [55]. Another major distinction from the intracellular environment is the high prevalence of nonenzymatic glycation in the extracellular space. Both ageing and hyperglycaemia are associated with accelerated extracellular glycation of lysine and arginine residues by reactions with glucose and its oxidation by‐products, leading to the build‐up of advanced glycation end products (AGEs) [56]. This is particularly detrimental to long‐lived structural proteins, such as types of collagens, that accumulate extensive damage over time due to the abundance of AGE crosslink glucosepane [57, 58]. The resulting stiffening of the ECM compromises wound healing and vascular elasticity during ageing, while also disrupting mechanotransduction and ultimately contributing to the loss of cellular homeostasis [22, 59].

In Alzheimer's disease (AD), AGE modifications are enriched in amyloid plaques and accelerate Aβ aggregation [60]. The mild acidification of extracellular fluids, including the blood, cerebrospinal fluid (CSF) and lymph, is another age‐related change impacting the extracellular proteome [61, 62, 63, 64]. The pH of the human brain and that of C57BL/6 mice decreases with age, and a similar acidosis is also observed in the postmortem brain and CSF samples of AD patients [61]. Notably, accelerated acidosis impairs primary microglia uptake of Aβ and is associated with increased *in vivo* Aβ plaque load in the APP‐PS1 mouse model of AD [61]. In contrast, alkalotic treatment has been shown to extend lifespan in wild‐type *Drosophila* [62, 64]. Together, this suggests an age‐dependent, pH‐mediated dysregulation of the extracellular proteostasis. Moreover, ageing significantly impairs temperature homeostasis through the blunting of hypothalamic thresholds, attenuated vasodilation and sweating, and the progressive loss of brown adipose tissue thermogenesis [65, 66, 67]. This systemic failure to buffer against thermal fluctuations creates a thermodynamically unstable environment that may facilitate protein aggregation in the extracellular space.

## Changes in fluid dynamics with age

Circulation between extracellular compartments is normally highly dynamic, ensuring the exchange of water and small solutes between plasma and interstitial fluids and enabling the removal of waste products through the lymphatic system. Shear stress generated by continuous blood flow imposes significant biophysical strain, destabilising extracellular proteins and enhancing their aggregation [68]. Such haemodynamic forces on arterial walls have been implicated in atherosclerosis, characterised by pathological amyloid‐like protein deposits. Basement membrane thickening along the microvasculature, due to increased ECM deposition and AGE modifications, is associated with altered capillary mechanics and impaired blood flow [69]. Moreover, ageing compromises interstitial fluid clearance as lymphatic vessel contractility declines with the loss of lymphatic muscle cells [70]. In the brain, age‐related disruption in glymphatic function disrupts the flow of CSF into the brain and the subsequent clearance of extracellular proteins from the parenchymal interstitial fluid into perivenous drainage pathways [71, 72, 73, 74, 75]. This initiates a self‐amplifying cascade in which aggregation‐prone proteins, such as Aβ accumulate around capillaries, further obstructing drainage and possibly accelerating AD progression [76, 77]. Together, these observations demonstrate that efficient fluid exchange across extracellular compartments is critical to maintain extracellular proteostasis and its age‐related decline directly contributes to extracellular protein aggregation (Fig. 1).

## Model systems to study extracellular protein damage and its age‐related regulation

An overview of model systems is presented in Table 1. Research into extracellular protein aggregation has been greatly advanced through transgenic mouse models that overexpress human aggregation‐prone proteins, including mutant APP generating high levels of Aβ, PrP and hIAPP. These models have provided critical insights into the dynamics of extracellular aggregation, including seeding mechanisms and pathology propagation. However, ageing remains the predominant risk factor for sporadic neurodegenerative disorders, and a major limitation of these models is their reliance on ectopic overexpression of mutant proteins in relatively young animals [78]. Surprisingly, despite the substantial aggregation burden, these transgenic mice rarely exhibit the extensive brain atrophy and neuronal loss characteristic of human neurodegenerative diseases. Although wild‐type mice display increased age‐dependent protein insolubility [79], synaptic decline [80, 81] and cognitive impairment [82], the extent of brain volume loss with age is less pronounced compared with humans [83, 84]. Despite these caveats, extracellular aggregation in mouse models has led to valuable insights into the roles of extracellular chaperones (ECs) and proteases [85, 86, 87, 88]. Studying ECM integrity is an important readout to assess ECM homeostasis and its protein quality control (PQC) with age [18]. The combined quantification of collagen deposition and *ex vivo* tail tendon breakage [89, 90, 91] is an effective method to assess damage by AGEs and impaired remodelling with age. Overall, further work examining extracellular proteostasis in physiologically ageing mice, either in the context of disease‐associated protein expression or independent of disease processes, is required to elucidate extracellular PQC mechanisms and assess capacity to mitigate protein damage during ageing.

**Table 1:** Experimental models used to study extracellular proteostasis. Summary of major *in vivo*, *ex vivo* and *in vitro* models used to investigate extracellular protein damage and the extracellular proteostasis network. Each system provides complementary strengths for studying aggregation mechanisms, extracellular chaperone and protease function, ECM remodelling and aggregate clearance, while differing in physiological relevance and ability to capture ageing‐associated changes.

Model system	Key advantages	Key limitations	Best suited for
Transgenic mouse models (e.g. APP, PrP and hIAPP)	Physiological tissue context Enables study of aggregation *in vivo* Genetic manipulation of exPN components	Often rely on overexpression of mutant proteins Limited ageing context Weak neurodegeneration compared with humans	Aggregation mechanisms *In vivo* validation of ECs/proteases Therapeutic testing
Wild‐type ageing mice	Natural ageing context No overexpression artefacts Enables study of baseline extracellular proteostasis decline	Slow ageing Mild phenotype Limited aggregation burden	Age‐dependent extracellular proteostasis decline ECM remodelling Clearance pathways
*Caenorhabditis elegans*	Short lifespan High genetic tractability Whole‐organism extracellular proteostasis analysis *In vivo* aggregation reporters	Simplified physiology Limited tissue complexity ECM differs from mammals	Discovery of exPN factors System‐level extracellular proteostasis control Genetic screening
*Drosophila melanogaster*	Rapid ageing Tissue‐specific expression Genetic tools	Less ECM complexity than mammals Limited extracellular compartment diversity	Neurodegeneration Functional effects of ECs
*In vitro* aggregation assays (ThT, turbidity)	High control over conditions Quantitative kinetics Mechanistic dissection Easy testing of ECs	No cellular context Oversimplified environment No clearance mechanisms	Aggregation kinetics Mechanisms of EC action Physicochemical effects
Body fluids (Plasma, CSF)	Direct human relevance Biomarker discovery Longitudinal analysis possible	Limited mechanistic insight Complex mixture Indirect readouts	Aggregate detection exPN factor levels Clinical correlations
Tissue explant‐derived organoids	Native human tissue Preserves existing pathology and aged state Relevant ECM environment	Limited availability High variability between donors Often short‐term experiments	Human‐specific aggregation Ageing studies Disease relevance
Primary cell‐derived organoids	Preserves human donor‐specific features, including some ageing aspects Defined, controllable composition Expandability Tunable ECM	Limited availability Variability between donors Limited cell diversity, bias towards proliferative cells Sensitive to culture conditions	Human‐specific mechanisms Ageing studies Disease mechanisms *Ex vivo* screening
iPSC‐derived organoids	Human genetic background Controlled manipulation Scalable	Immature tissue state Very limited ageing features Lack of vasculature/immune system	Human‐specific mechanisms Gene function studies
3D biomaterial scaffolds model	Controllable environment Synthetic ECM available Tunable matrix stiffness and composition Easy manipulation High reproducibility Manipulation of AGE crosslinks possible	Artificial environment Variability of natural biomaterials Lack of cellular complexity Simplified architecture compared with tissue	ECM remodelling ECM‐driven aggregation studies Physicochemical effects Drug screening

APP, amyloid precursor protein; PrP, prion protein; hIAPP, human islet amyloid polypeptide; ThT, thioflavin‐T; CSF, cerebrospinal fluid; iPSC, induced pluripotent stem cells; EC, extracellular chaperone; ECM, extracellular matrix; ExPN, extracellular proteostasis network.

Short‐lived organisms such as *C. elegans* and *Drosophila*, despite their phylogenetic distance from mammals, offer powerful models for investigating extracellular proteostasis due to their rapid ageing and genetic tractability. In *C. elegans*, proteomic identification of secreted proteins with high aggregation propensity in aged animals has enabled the development of transgenic strains overexpressing fluorescently tagged proteins to track extracellular aggregation *in vivo*. These models have facilitated systematic mapping of the exPN through RNAi screening, leading to the discovery of the first EC in the nematode [92]. *C. elegans* also offers a wide range of *in vivo* models for ECM misfolding and turnover with age, including real‐time monitoring of collagen turnover with photoswitchable fluorescent tags and stiffness quantification through fluorescence resonance energy transfer (FRET) measurements [93, 94]. While less complex than mammalian systems, these models are uniquely suited for exploring organismal extracellular proteostasis regulation and for discovering novel extracellular PQC factors that may be conserved across species.

*In vitro* experiments provide additional mechanistic insight into the process of extracellular aggregation and its regulation. Test‐tube aggregation assays, monitored by Thioflavin‐T (ThT) fluorescence or turbidity, provide quantitative readouts of amyloid or amorphous protein aggregation and enable the assessment of how extracellular PQC factors or changes in physicochemical conditions modulate this process. Extracellular aggregation of neurodegenerative disease‐related proteins has been successfully modelled in organotypic mouse brain slice cultures over several weeks by adding exogenous aggregate seeds kept in culture [95, 96]. Although these slices are derived from juvenile or neonatal stages, the proteotoxic stress caused by the aggregation process induces gene expression changes reminiscent of those observed during ageing [97]. Given the fundamental differences between mouse and human, developing *in vitro* human models is essential to gain deeper insights into the process of extracellular protein aggregation and evaluate the efficacy of extracellular PQC factors. Human tissue explants obtained from elective surgery or postmortem sources are particularly valuable, as they can be maintained *in vitro* as organoid cultures. Those derived from aged donors are especially useful models for studying ageing and extracellular proteostasis, including ECM‐remodelling dynamics within a physiologically relevant context [98, 99]. For example, organotypic brain slice cultures obtained from aged individuals can be successfully maintained *ex vivo* over several weeks in culture and pre‐existing amyloid plaques remain stable over time [100, 101]. Alternatively, organoids can be reconstituted through expansion of proliferative primary cells, allowing for greater experimental control, while retaining some ageing aspects of the tissue of origin. Human iPSC‐derived cerebral organoids offer a more accessible alternative to tissue‐derived organoids, particularly for hard‐to‐obtain brain and cardiac tissues. However, although these organoids can model AD‐related amyloid features, including the influence of organoid‐secreted ECM on pathology [102], they often lack mature phenotypes, with transcriptional profiles resembling prenatal stages [102, 103, 104, 105]. Furthermore, biomaterial scaffold‐based 3D systems, including hydrogel and ECM‐mimetic culture platforms, provide controlled extracellular environments in which matrix stiffness, composition and diffusion properties can be experimentally manipulated [106]. Such models are increasingly being utilised to investigate how ECM architecture influences aggregate formation, propagation and clearance [107, 108, 109]. Together, these emerging systems help bridge the gap between *in vitro* and complex *in vivo* models, while incorporating human‐specific aspects.

Studying body fluids offers a powerful approach to understanding human extracellular proteostasis, allowing the monitoring of protein aggregate levels in relation to age and health status, while also providing an important starting point for identifying novel extracellular regulators that bind misfolded proteins [110].

## Extracellular PQC mechanisms

The most recent US Proteostasis Consortium analysis identifies >3100 components in the human PN, yet only 23 act extracellularly, either as chaperones or proteases, underscoring the relative scarcity of mechanisms dedicated to extracellular proteostasis (www.proteostasisconsortium.com/pn‐annotation/). Investigations in *C. elegans* have identified 57 previously unrecognised extracellular regulators of aggregation, indicating that the exPN is far more complex than previously appreciated [92]. These regulators display a diverse range of predicted structural folds, and studying them and their human orthologs is likely to uncover novel mechanisms contributing to extracellular proteostasis.

## Extracellular chaperones

Because of the low levels of ATP, chaperones outside cells are known to act mainly as holdases by binding to misfolded proteins to prevent their aggregation, rather than engaging in refolding activity. Several intracellular chaperones can be released into the extracellular space through noncanonical secretion pathways. While intracellular chaperones such as HSP70 and small heat shock proteins (sHSPs) have been shown to mitigate extracellular protein‐aggregation toxicity, their effects appear to be largely local and context‐dependent [111, 112]. In many cases, their extracellular presence reflects stress signalling rather than a dedicated proteostasis function, particularly in inflammatory or cancer settings [113]. Intracellular chaperones, such as HSP40 and HSPB1, actively transferred between cells, play an important role in enhancing the recipient cell's intracellular proteostasis capacity [114, 115]. In contrast, there is clear evidence for a *bona fide* extracellular chaperone role of HSP90 in the ECM, where it binds to various clients, such as fibronectin and matrix metalloproteinase 2 (MMP2), to influence their stability [7, 116]. Relevant to ECM function, the endoplasmic reticulum (ER) resident protein disulfide isomerase (PDI) can be secreted into the ECM, where it regulates integrin‐mediated cellular adhesion through both its disulfide‐exchange activity and chaperone holdase activities [117, 118, 119]. During ER stress, ER chaperones play a prominent role in extracellular PQC, as reviewed by Mesgarzadeh *et al*. [120]. A well‐characterised example is the HSP40 co‐chaperone ERdj3/DNAJB11, which is co‐secreted with aggregation‐prone proteins during ER stress and helps protect both the ER lumen and the extracellular environment by preventing further aggregation [120, 121]. These intracellular chaperones likely act only at a local level to support extracellular proteostasis. In contrast, large‐scale and systemic control of extracellular protein misfolding relies on distinct ECs. The US Proteostasis consortium survey annotates 22 ECs in humans, including 5 newly discovered proteins with antiaggregation activity [110]. These are mostly multi‐functional proteins with holdase function. Most identified ECs inhibit both amyloid and amorphous aggregation, though they are less efficient against the latter. This is likely due to proportionally higher amounts of exposed hydrophobic regions that facilitate intermolecular interactions leading to amorphous aggregation. Consequently, higher chaperone concentrations are required to effectively counteract this amorphous aggregation process.

**Fig. 2:**
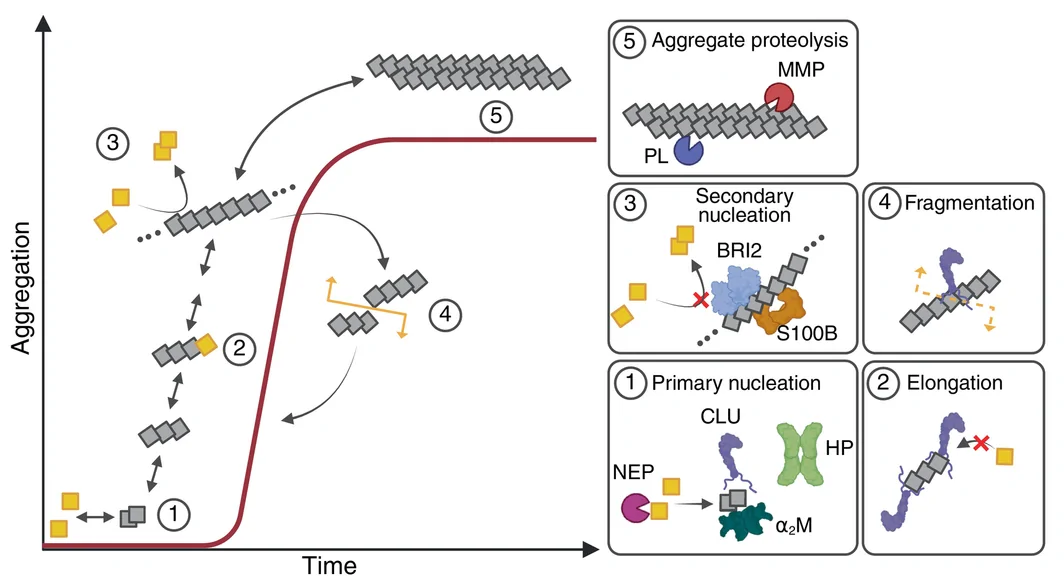
Extracellular proteostasis mechanisms targeting the amyloid aggregation process. Schematic illustrating the kinetics of amyloid formation and the stages at which extracellular proteostasis mechanisms intervene. Amyloid formation begins with an initial lag phase, during which monomers nucleate and form oligomeric species, which are recognised and suppressed by ECs CLU, α2M and HP, as well as extracellular protease NEP (box 1). During the fibril growth phase, elongation via monomer addition at fibril or oligomer ends is inhibited by CLU (box 2), while secondary nucleation from mature fibrils is prevented by ECs BRI2 and S100B (box 3). CLU also stabilises fibrils against fragmentation (box 4). Aggregation plateaus once monomers are depleted. In later stages, extracellular proteases such as plasmin and MMPs cleave compact amyloid fibrils, contributing to their clearance (box 5). BRI2, BRICHOS containing protein; CLU, clusterin; EC, extracellular chaperone; HP, haptoglobin; MMP, matrix metalloproteinase; NEP, neprilysin; PL, plasmin; α2M, α2‐macroglobulin. Created in BioRender. David, D. (2026) https://BioRender.com/kumgieq.

Insights from *in vitro* aggregation assays using ThT dye and mathematical modelling reveal that many ECs disrupt amyloid fibril formation at substoichiometric ratios by targeting the early phases of aggregation (Fig. 2). One of the most well‐studied EC, clusterin, has a broad range of both amyloidogenic and amorphous aggregating substrates [122]. Clusterin inhibits the elongation stage of the aggregation process at low substoichiometric levels (1:100), by binding to the ends of Aβ fibrils and oligomer/prefibrils to stop addition of monomers [123]. Clusterin can also inhibit fragmentation of other substrates, such as β‐2‐microglobulin through interactions with the fibrils [124]. Recent findings highlight that the unstructured tail regions of the clusterin α‐ and β‐chain complex play a critical role in preventing protein aggregation [125]. These hydrophobic tails share a compositional bias with the N‐terminal regions of sHSPs, which are essential for chaperone activity. Remarkably, Yuste‐Checa *et al*. demonstrated that grafting these clusterin tails onto GFP is sufficient to confer chaperone function, rendering the modified GFP nearly as effective as full‐length clusterin in inhibiting aggregation [125]. α_2_‐macroglobulin and haptoglobin, primarily recognised for their roles as a protease inhibitor and haemoglobin binder, respectively, also exhibit EC activity, inhibiting aggregation through interactions with oligomeric species [126]. Secondary nucleation of amyloid fibrils (i.e. surface‐catalysed nucleation) is effectively suppressed by both S100B and BRICHOS domains [127, 128]. S100B, an abundant soluble protein in the brain, binds to Aβ as a dimer through an electrostatic interaction within its dimer cleft [129]. BRICHOS domains, present in 10 human protein families, share a conserved structural fold despite diverse primary sequences [130]. Among these, BRICHOS domains from proSP‐C and Bri2 have been extensively characterised and shown to target fibril ‘hot spots’, selectively inhibiting secondary nucleation [128]. Unlike proSP‐C, Bri2 is widely expressed, and proteolytic processing of membrane‐bound Bri2 releases a soluble BRICHOS domain into the extracellular space, suggesting an active role in extracellular proteostasis [131]. Intriguingly, under redundant conditions, Bri2 BRICHOS domains can oligomerise, acquiring holdase‐like activity that prevents amorphous aggregation [132]. Interestingly, neuroserpin (NS) and transthyretin (TTR) exhibit specificity against amyloid aggregation, a property attributed to an amphipathic α‐helix critical for their inhibitory function [133]. Notably, NS redirects Aβ into an off‐pathway, nontoxic aggregation route, reducing its pathogenic potential [134]. Although the extracellular proteostasis machinery within the ECM remains relatively poorly characterised, emerging evidence suggests that SPARC (Secreted Protein, Acidic, Rich in Cysteine) functions as an EC. In invertebrates, SPARC maintains collagen solubility in the extracellular space, enabling its trafficking along basement membranes for polymerisation at distal sites [135]. In mammals, SPARC appears to play a similar role and patients carrying missense mutations in SPARC exhibit increased tendon injuries and impaired collagen type I maturation *in vitro* [136]. Overall, by acting on different aggregation‐prone substrates and intervening at distinct stages of the amyloid formation pathway, ECs can cooperate to preserve the integrity of the extracellular proteome. A clear example of this synergistic effect was observed *in vitro*, where clusterin and the proSP‐C BRICHOS domain jointly provide enhanced protection against Aβ aggregation by targeting sequential steps in the aggregation process (Fig. 2) [123].

ECs help limit protein aggregation, but because ECs lack refolding activity, damaged proteins ultimately require removal through cellular uptake. In mammals, the liver and kidney are major sites for clearing circulating misfolded proteins, and most plasma proteins are rapidly turned over (e.g. >20 days in humans) [137, 138]. Aggregation‐prone proteins, however, are cleared more slowly, as observed in *C. elegans*, where secreted GFP is rapidly removed while aggregating lipid binding protein 2 (LBP‐2) accumulates in the extracellular body cavity [92]. Clearance efficiency is enhanced by circulating glycosidases, which remodel N‐glycans on secreted proteins to promote recognition by lectin receptors and subsequent endocytosis [139]. ECs such as clusterin and α_2_‐macroglobulin accelerate the intracellular degradation of damaged proteins through binding and promoting receptor‐mediated uptake [4, 5]. Intriguingly, heparan sulfate proteoglycans act as receptors for clusterin‐substrate complexes, potentially enabling a wide range of cell types to contribute to the clearance of extracellular misfolded proteins [140].

## Extracellular proteases

Extracellular proteases are another key component of the extracellular PQC arsenal. Extracellular proteolysis contributes to preventing aggregate formation and to dismantling existing large aggregate deposits. These actions work in synergy with ECs to enable extracellular proteostasis. One of the best‐characterised extracellular proteolytic systems is fibrinolysis, in which plasmin, generated by tissue‐type plasminogen activator (tPA) activation of plasminogen, degrades the aggregates. Its primary function is to dissolve blood clots during wound healing by cleaving fibrin polymers. Beyond its canonical role in clot breakdown, the tPA/plasmin system targets a wide range of aggregation‐prone substrates, including Aβ, PrP and IAPP, while also displaying activity against amorphous aggregates [141, 142, 143, 144]. Mechanistically, tPA and plasminogen are recruited by lysine residues exposed during fibrin polymerisation and during the unfolding events characteristic of amorphous protein aggregation. This spatial arrangement facilitates tPA‐mediated conversion of plasminogen into plasmin, which subsequently degrades the fibrin matrix [145]. In disease settings, the amyloid cross‐β sheet structure, characteristic of pathological fibrin aggregates and disease‐associated aggregates, strongly recruits tPA/plasminogen to trigger subsequent plasmin cleavage of amyloid fibrils [143]. This process is likely critical for clearing amyloid deposits that accumulate with age and disease [39, 146]. *In vitro* data imply that ECs such as clusterin could act synergistically with the tPA/plasmin system *in vivo*, by specifically binding the proteolytic fragments generated during aggregate degradation, thus suppressing their downstream toxicity [141]. Among extracellular proteases, neprilysin, a zinc‐metalloprotease present in both membrane‐bound and soluble secreted forms, is another potential key extracellular PQC factor and is estimated to mediate roughly half of all Aβ clearance in the brain [147]. Although it is largely ineffective at degrading preformed aggregates, neprilysin efficiently limits the formation of pathological Aβ assemblies by cleaving monomeric and oligomeric Aβ species (Fig. 2) [147, 148]. Blocking zinc‐metalloproteases neprilysin and endothelin‐converting enzymes leads to a loss of Aβ homeostasis and a > 5‐fold rise in cerebral Aβ in wild‐type mice [149]. Matrix Metalloproteinases (MMPs) are another important player in extracellular PQC. These zinc‐dependent endopeptidases are secreted into the ECM, where they degrade proteins and can also cleave misfolded proteins or amyloid fibrils both in and outside the ECM, facilitating their removal. MMP1 is known to degrade functionally aggregating proteins, such as aggrecan, perlecan, nidogen, serpins and versican [150], while MMP13 degrades aggrecan and perlecan [151]. Additionally, MMP1, enabled by its haemopexin‐like domain, can unwind fibrillar collagen, together with MMP8 and MMP13, which then allows MMP2 and MMP9 to bind to these precleaved fibrils and continue their unwinding and degradation [152, 153, 154, 155]. MMP‐mediated ECM remodelling is controlled by ECs. For example, extracellular HSP90 regulates ECM degradation by forming a chaperone complex with tissue inhibitor of metalloproteinases 2 (TIMP2), which inhibits MMP2 [7, 156, 157]. Only after displacement of TIMP2 is MMP2 reactivated and can proceed to cleave collagen [7]. Similarly, clusterin inhibits MMP9 activity by binding to its catalytic domain to avoid overactivation of MMP9 and reduce inflammatory responses [158]. AGE‐modified collagen increasingly resists MMP cleavage with age, causing dysregulated ECM turnover and the emergence of stiff, crosslinked domains interspersed with weaker, fragmented ECM regions [8]. Our current understanding of how MMPs degrade pathological extracellular protein aggregates largely stems from work on Aβ. MMP9 and MMP2 sequentially cleave Aβ into soluble, nontoxic fragments found in the CSF to promote clearance [159]. Evidence with aged AD mouse brain slices shows that MMP9 can degrade compact amyloid plaques [160]. Oxidation is a potent inducer of MMPs expression and proteolytic activity [161, 162, 163]. Metalloproteases may also indirectly influence protein aggregation. ECM components are incorporated into AD amyloid plaques and a COL25A1 fragment enhances plaque compaction and local toxicity [164]. An RNAi screen in *C. elegans* further demonstrated that modulating ECM and remodelling factors, most notably the ADAM‐9 ortholog, alters Aβ aggregation and facilitates deposit clearance [165]. Together, these proteases likely contribute to physiological clearance mechanisms that become insufficient with age.

## ExPN deregulation with age and disease

**Fig. 3:**
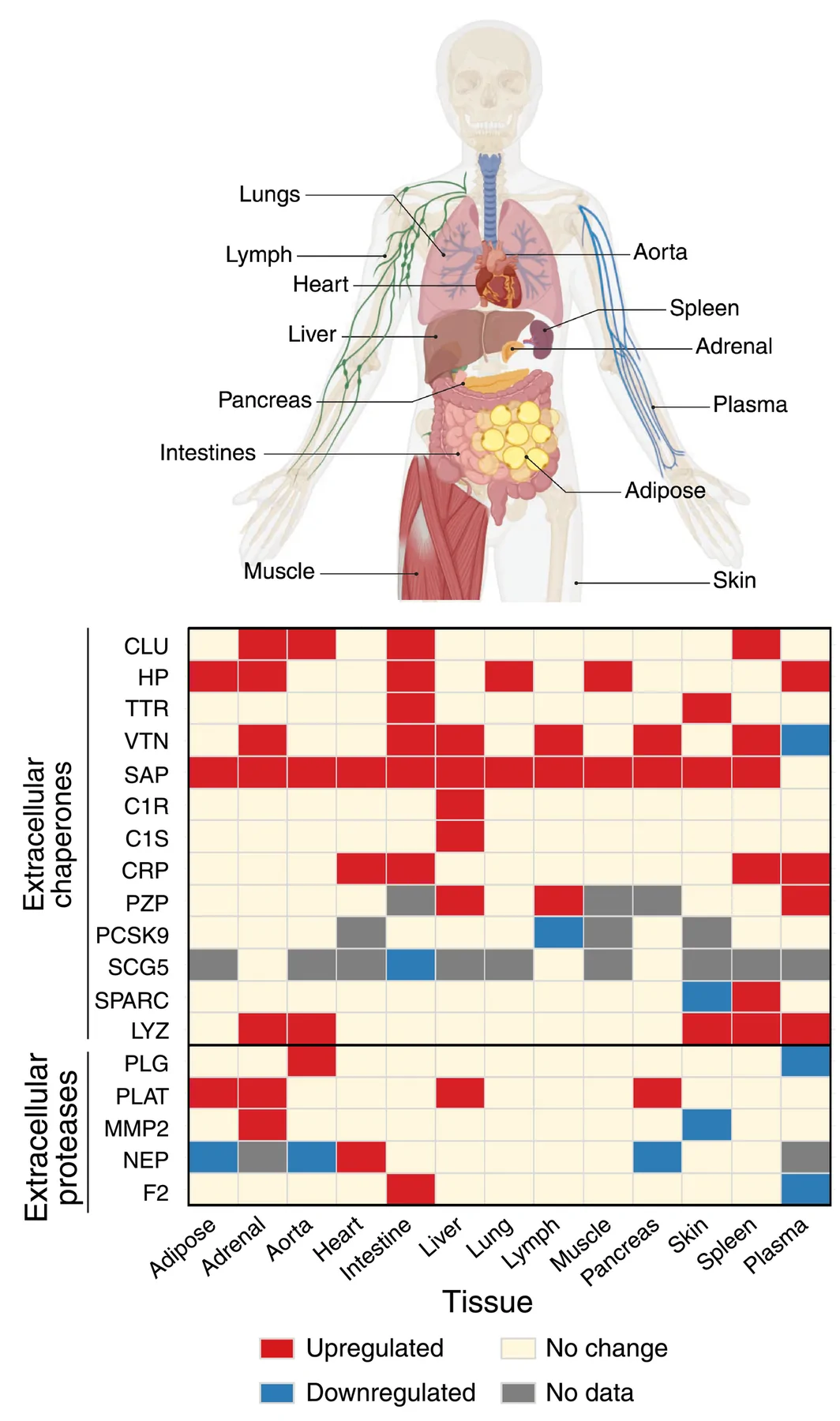
Human tissue‐specific, age‐dependent changes in the levels of extracellular chaperones and proteases. Publicly available proteomics datasets (IPX0010296000) [15, 167] were used to calculate the tissue‐specific changes in the levels of secreted extracellular chaperones and proteases (exPN factors) with age. The heatmap depicts changes in levels of the extracellular chaperones and proteases with age in each tissue (*P* < 0.05). exPN factors were compiled from the literature and the US Proteostasis Consortium annotation, and only those with a signal peptide were considered. For calculating changes in all tissues except the plasma, a nonparametric Mann–Whitney U‐test was performed between the older and the younger group to determine statistical significance. For calculating changes in the plasma, the ageing plasma proteome tool (twc‐stanford.shinyapps.io/aging_plasma_proteome/) [167] was used. C1R, complement C1r; C1S, complement C1s; CLU, clusterin; CRP, C‐reactive protein; F2, coagulation factor II thrombin; HP, haptoglobin; LYZ, lysozyme; MMP2, matrix metalloproteinase 2; NEP, neprilysin; PCSK9, proprotein convertase subtilisin/kexin type 9; PLAT, tissue‐type plasminogen activator 3; PLG, plasminogen; PZP, pregnancy zone protein; SAP, serum amyloid P component; SPARC, secreted protein acidic and rich in cysteine; TTR, transthyretin; VTN, vitronectin. Created in BioRender. David, D. (2026) https://BioRender.com/0hb1mu9.

Intracellular PN is dysregulated and overwhelmed with age leading to proteostasis loss and accumulation of protein aggregates [166]. In *C*. *elegans*, levels of exPN components are deregulated with age [92]. To obtain an overview of age‐related changes in the human exPN, we evaluated abundance levels of 18 extracellular PQC factors in plasma and tissues using publicly available proteomic datasets [15, 167]. Lehallier *et al*. measured the levels of nearly 3000 proteins in the plasma of over 4000 young adults to nonagenarians (18–95) and developed a new bioinformatics approach that uncovered nonlinear alterations in the human plasma proteome with age (twc‐stanford.shinyapps.io/aging_plasma_proteome/) [167]. Ding *et al*. identified age‐related changes across tissues by comparing older individuals (45–68 years) to a younger group (14–30 years) [15]. The most pronounced age‐related changes in exPN were found in the intestine, adrenal gland, liver, spleen and aorta, where at least five extracellular PQC factors showed altered abundance, largely exhibiting age‐associated increases (Fig. 3, Table 2). In plasma, more than one third of detected extracellular PQC factors also changed with age but showed a more heterogeneous pattern than in tissues, with both increases and decreases in abundance. Across multiple tissues, SAP, vitronectin and haptoglobin show the most consistent age‐associated increases. By contrast, only a few extracellular PQC factors showed age‐related decreases, and neprilysin was the only protein reduced across three tissues (aorta, adipose tissue and pancreas), while displaying higher levels in the heart. Together with evidence of declining brain neprilysin levels after middle age [168], these findings suggest that reduced neprilysin may be a major contributor to the age‐associated accumulation of extracellular protein aggregates. Overall, the functional implications of increased levels of extracellular PQC factors with age remain unclear. Further studies are needed to determine whether these factors can be effective in rebalancing extracellular proteostasis in an ageing context. For instance, tPA levels decline with age while plasminogen activator inhibitor‐1 (PAI‐1) increases, shifting plasminogen processing towards reduced plasmin production and thus potentially impaired clearance of Aβ and other aggregates [169, 170]. Furthermore, the widespread accumulation of SAP is likely reflective of its irreversible incorporation into aggregates, where it remains trapped, unavailable to promote cellular uptake of damaged proteins.

**Table 2:** Human tissue‐specific, age‐dependent changes in the levels of extracellular chaperones and proteases. Publicly available proteomics datasets (IPX0010296000) [15] were used to calculate the tissue‐specific changes in the levels of extracellular chaperones and proteases between older (45–68 years) and younger individuals (14–30 years). ExPN factors were compiled from the literature and the US Proteostasis Consortium annotation. Only exPN factors with a signal peptide were considered. A nonparametric Mann–Whitney U‐test was performed between the older and the younger group to determine statistical significance.

Tissue	Protein	Symbol	Level with age	*P*‐value	Mean abundance in young	Mean abundance in aged
Adipose	Haptoglobin	HP	Up	0.0343	21.17	22.83
	Neprilysin	NEP	Down	0.0343	17.95	17.45
	Tissue‐type plasminogen activator 3	PLAT	Up	0.0231	15.96	16.56
	Serum amyloid P component	SAP	Up	0.0312	21.60	22.25
Adrenal	Clusterin	CLU	Up	0.035	20.80	20.96
	Haptoglobin	HP	Up	0.0368	21.64	22.19
	Lysozyme	LYZ	Up	0.00381	17.83	18.67
	Matrix metalloproteinase 2	MMP2	Up	0.00332	15.39	15.85
	Tissue‐type plasminogen activator 3	PLAT	Up	0.0197	15.15	15.57
	Serum amyloid P component	SAP	Up	0.0000723	21.16	22.03
	Vitronectin	VTN	Up	0.00531	20.19	20.75
Aorta	Clusterin	CLU	Up	0.00261	23.29	24.43
	Lysozyme	LYZ	Up	0.000654	19.81	21.53
	Neprilysin	NEP	Down	0.0248	14.64	11.21
	Plasminogen	PLG	Up	0.0464	20.82	21.42
	Serum amyloid P component	SAP	Up	0.00131	23.34	24.94
Heart	C‐reactive protein	CRP	Up	0.0424	19.47	20.51
	Neprilysin	NEP	Up	0.0145	14.81	15.38
	Serum amyloid P component	SAP	Up	0.000547	20.75	21.55
Intestine	Clusterin	CLU	Up	0.0104	20.02	20.78
	C‐reactive protein	CRP	Up	0.0424	17.56	19.19
	Coagulation factor II, thrombin	F2	Up	0.0194	17.27	18.33
	Haptoglobin	HP	Up	0.0104	20.18	22.70
	Serum amyloid P component	SAP	Up	0.000547	19.75	21.82
	Secretogranin V	SCG5	Down	0.0145	16.25	15.02
	Transthyretin	TTR	Up	0.0145	19.24	20.06
	Vitronectin	VTN	Up	0.00191	19.38	20.46
Liver	Complement C1r	C1R	Up	0.01295	19.01	19.18
	Complement C1s	C1S	Up	0.035	19.94	20.16
	Tissue‐type plasminogen activator 3	PLAT	Up	0.000556	11.91	13.43
	Pregnancy zone protein	PZP	Up	0.0247	15.17	16.94
	Serum amyloid P component	SAP	Up	0.00147	21.40	22.25
	Vitronectin	VTN	Up	0.0000374	20.68	21.05
Lung	Haptoglobin	HP	Up	0.0303	21.40	23.79
	Serum amyloid P component	SAP	Up	0.0303	20.92	23.02
Lymph	Proprotein convertase subtilisin/kexin type 9	PCSK9	Down	0.0433	14.25	13.66
	Pregnancy zone protein	PZP	Up	0.012	11.25	14.98
	Serum amyloid P component	SAP	Up	0.00401	21.99	23.04
	Vitronectin	VTN	Up	0.012	20.47	21.25
Muscle	Haptoglobin	HP	Up	0.000707	19.33	22.23
	Serum amyloid P component	SAP	Up	0.0261	18.78	19.68
Pancreas	Neprilysin	NEP	Down	0.0332	13.97	12.09
	Tissue‐type plasminogen activator 3	PLAT	Up	0.0387	13.85	14.17
	Serum amyloid P component	SAP	Up	2.58E‐08	18.53	20.52
	Vitronectin	VTN	Up	0.000442	19.28	20.04
Skin	Lysozyme	LYZ	Up	0.000244	18.83	20.10
	Matrix metalloproteinase 2	MMP2	Down	0.0341	19.45	18.93
	Serum amyloid P component	SAP	Up	0.000699	22.05	23.88
	SPARC	SPARC	Down	0.0135	17.61	17.13
	Transthyretin	TTR	Up	0.0134	21.47	21.95
Spleen	Clusterin	CLU	Up	0.00659	20.68	21.30
	C‐reactive protein	CRP	Up	0.0229	18.93	20.49
	Lysozyme	LYZ	Up	0.0326	22.19	22.75
	Serum amyloid P component	SAP	Up	0.0000814	21.16	22.45
	SPARC	SPARC	Up	0.0128	18.55	19.40
	Vitronectin	VTN	Up	0.00401	20.50	21.78

Growing evidence implies that age‐related dysregulation of exPN components contributes to neurodegenerative disease pathology [171]. ECs such as clusterin [172], SAP protein [173], α_2_‐macroglobulin [174] and haptoglobins [175] are found to be associated/co‐localised with Aβ plaques *in vivo*, highlighting their potential involvement in AD pathology. These ECs are also elevated in CSF, brain, and the plasma of patients with AD [176, 177, 178]. Studies examining clusterin levels in AD report inconsistent findings, with some detecting increases and others decreases in CSF or plasma, likely reflecting stage‐dependent changes linked to disease progression and ageing [179, 180, 181, 182]. Beyond age‐related changes in abundance, several ECs and proteases have been genetically linked to age‐associated pathologies. The clusterin gene is the third most significant genetic risk factor for sporadic AD [183, 184, 185, 186], including variants that reduce its secretion and trigger alternative splicing [187, 188]. Clusterin variants are also linked to differences in cognition, brain activity and structure in healthy individuals, which could influence vulnerability to AD [189, 190, 191]. Polymorphisms in S100B are associated with changes in CSF levels of tau/Aβ ratios [192]. A variant of complement C1s was identified in a trans‐ethnic GWAS analysis as potentially contributing to AD risk in the Japanese population [193]. Genetic evidence further supports the protective role of extracellular proteases in contributing to the clearance of protein aggregation. MMPs dysregulation is implicated in disorders featuring extracellular protein aggregation and fibrosis [194, 195, 196]. Polymorphisms in MMP1, MMP2, MMP3 and MMP9 are linked to elevated risk of AD and vascular dementia [197, 198]. Risk variants in MME, which encodes neprilysin, have been identified as contributing to sporadic AD, including a missense mutation reducing its ability to degrade Aβ by impairing secretion of neprilysin [147, 199, 200]. While available genetic studies implicate the exPN in influencing pathological extracellular aggregation in AD, more studies are needed to explore genetic associations between exPN genes and other extracellular aggregation‐associated disorders, including systemic amyloidosis.

Taken together, the observed age and disease‐associated shifts in exPN abundance, along with supporting genetic evidence, highlight the relevance of exPN in sustaining extracellular proteostasis during ageing. Increased levels of many extracellular PQC factors with age and AD further support the notion that the exPN is induced by proteostress to counteract the accumulation of extracellular aggregates [120]. Importantly, given that extracellular PQC factors also perform many nonproteostatic functions, it is essential to consider how their additional roles are impacted by and contribute to age or disease‐related alterations.

## Extracellular proteostasis and the immune system

**Fig. 4:**
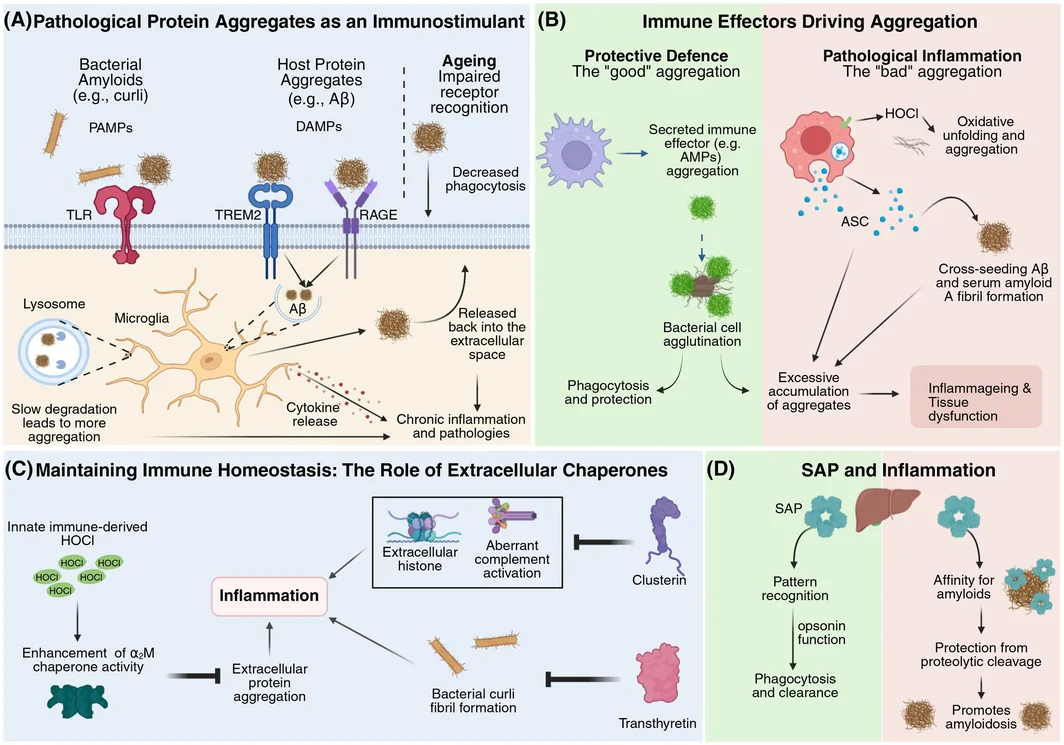
Crosstalk between extracellular proteostasis and inflammation. (A) Schematic representation of extracellular protein aggregates as an immunostimulant. Bacterial amyloids such as curli (recognised as PAMPs) and host protein aggregates (recognised as DAMPs), such as extracellular Aβ activate TLR, TREM2 and RAGE signalling pathways. Subsequently, aggregates are phagocytosed and destined for degradation in the lysosome. Slow lysosomal degradation can result in aggregate accumulation and potentially release back into the extracellular space. Notably, ageing is associated with impaired receptor‐mediated recognition of these aggregates, leading to decreased phagocytic clearance. Aggregates stimulate release of cytokines by immune cells, further driving chronic inflammation. (B) Schematic representation of how immune cells could contribute to aggregation in the extracellular space. Protective defence through aggregation of immune effectors around pathogens facilitates their phagocytosis and degradation. HOCl secreted by immune cells to neutralise pathogens can also lead to pathological aggregation by causing oxidative unfolding and aggregation of host proteins. Moreover, the intracellular ASC inflammasome complex leaking into the extracellular space cross‐seeds aggregation of host proteins. (C) Roles of extracellular chaperones in maintaining immune homeostasis. Innate immune‐derived HOCl enhances α2M chaperone activity, which in turn prevents extracellular aggregation, thereby limiting sterile inflammation. Similarly, transthyretin chaperone activity inhibits bacterial curli aggregation. Clusterin prevents aberrant activation of the complement system and neutralises extracellular histones, mitigating inflammatory cascades. (D) SAP and inflammation. SAP interacts with various pathogenic agents and amyloid aggregates in the extracellular space through its pentraxin domain, and aids in their clearance by acting as an opsonin. However, owing to its affinity for amyloid aggregates, it can stabilise aggregates and protect them from proteolytic degradation, thereby promoting amyloidosis. AMPs, antimicrobial peptides; ASC, apoptosis‐associated speck‐like protein containing a caspase recruitment domain; Aβ, amyloid β; DAMPs, damage‐associated molecular patterns; HOCl, hypochlorite; PAMPs, pathogen‐associated molecular patterns; RAGE, receptor for advanced glycation end products; SAP, serum amyloid P component; TLR, toll‐like receptor; TREM2, triggering receptors expressed in myeloid 2; α2M, α2‐macroglobulin. Created in BioRender. David, D. (2026) https://BioRender.com/f2f26az.

There is a complex multifaceted relationship between the immune system, protein aggregation and extracellular proteostasis. Protein aggregates are highly immunostimulatory (Fig. 4A) [201, 202]. Bacterial amyloids (e.g. curli involved in biofilms) are recognised as pathogen‐associated molecular patterns (PAMPs) by the Toll‐like receptor (TLR) 2 [203]. Similarly, the immune system recognises host protein aggregates as damage‐associated molecular patterns (DAMPs). ECM Hyaluronan and proteoglycan link protein 2 (HAPLN2) aggregates induce microglial inflammatory responses both *in vitro* and *in vivo* [28]. Aβ activates the CD36‐TLR4‐TLR6 complex to induce pro‐inflammatory cytokine release [42] and Aβ aggregates bind several microglia receptors, including Triggering Receptor Expressed on Myeloid Cells 2 (TREM2) and the receptor for advanced glycation end product (RAGE), promoting their phagocytosis [6]. Although Aβ fibrils are transported into the lysosomes, they are slowly degraded in these compartments [204], leading to their accumulation within lysosomes and even their release back into the extracellular space, thereby propagating pathology. However, microglia may improve the clearance of extracellular aggregates by other mechanisms, such as releasing MMPs and by targeted lysosome exocytosis [205, 206]. With age, the recognition of aggregates and their phagocytosis by microglia is impaired [207, 208]. Intriguingly, there is increasing evidence that Aβ aggregation and more generally amyloid aggregation of antimicrobial peptides (AMPs) and proteins is a key aspect of the secretory immune defence against pathogens and mediates bacterial cell agglutination (Fig. 4B) [209, 210, 211, 212]. Immune cells fuel extracellular protein aggregation through the release of hypochlorous acid (HOCl) [213] and apoptosis‐associated speck‐like protein containing a caspase recruitment domain (ASC), which cross‐seeds Aβ and serum amyloid A fibril formation [214, 215]. While host AMP aggregation enhances protection against pathogens, excessive accumulation of immune protein aggregates can provoke sustained immune activation, ultimately accelerating inflammageing [216, 217, 218]. A notable example is systemic amyloidosis arising from aggregating immunoglobulin light chains, leading to persistent inflammation and tissue dysfunction [219].

Extracellular proteostasis is likely to play an important role in maintaining immune homeostasis (Fig. 4C). By suppressing protein aggregation, ECs and proteases raise the threshold required for aggregation to enter its exponential, self‐amplifying phase, thereby limiting the accumulation of immunoreactive aggregates that drive chronic inflammation. In *C. elegans*, the exPN is actively upregulated during immune activation, highlighting its central role in the host defence. In response to a pathogenic challenge, exPN components are induced through the same stress‐activated MAPK signalling pathway that regulates the secretory immune response [92]. This exPN activation effectively limits extracellular protein aggregation during infection and enhances organismal survival. In mammals, immune system activation can similarly enhance extracellular proteostasis capacity. HOCl produced during neutrophil extracellular trap (NET) formation inactivates trapped pathogens by oxidative unfolding and aggregation of pathogen proteins [213, 220]. At the same time, HOCl‐induced oxidative modifications increase the chaperone capacity of α_2_‐macroglobulin and can even convert abundant plasma proteins, such as serum albumin, into chaperone‐like holdases, thereby helping to minimise aggregation‐driven self‐damage in the vicinity of the infection [221, 222]. Clusterin also plays an important role in limiting inflammation‐driven tissue injury, especially by binding and neutralising extracellular histones [223] and by inhibiting the polymerisation of complement components into the membrane attack complex [224, 225, 226]. ECs such as TTR directly help the immune system by inhibiting functional bacterial curli fibrillogenesis [227].

Deregulation of immune homeostasis and the rise of chronic, low‐grade inflammation are pervasive hallmarks of ageing. Elevated levels of several ECs across tissues with age, including the spleen, may represent a compensatory response of the exPN to increased immune‐related damage. However, this upregulation is not universally beneficial. Some ECs possess intrinsic amyloidogenic potential or can promote aggregation under certain conditions. A notable example is SAP, predominantly produced by the liver and found to accumulate widely across tissues with age (Fig. 4D). SAP is a pentameric pattern‐recognition molecule highly upregulated during immune activation, where it functions as an opsonin to enhance phagocytosis of microbial targets [228, 229]. Its strong affinity for amyloids makes it a ubiquitous constituent of amyloid deposits. Although SAP can exhibit antiaggregation activity, it also stabilises aggregates by protecting them from proteolytic degradation, and *in vivo* studies indicate that SAP ultimately promotes amyloidosis [230, 231, 232, 233].

## Strategies to enhance extracellular proteostasis

Studies in *C. elegans* demonstrate that enhancing extracellular proteostasis, including maintaining ECM homeostasis, promotes longevity [92, 94]. A coordinated upregulation of exPN factors is likely to provide the most robust enhancement of extracellular proteostasis capacity. In response to a pathogenic insult, the stress‐activated MAPK signalling pathways, JNK‐1 and KGB‐1 in *C. elegans*, act as key regulators, driving exPN factor expression to suppress protein aggregation and extend *C. elegans* survival [92]. Longevity signalling through reduced insulin/IGF‐1‐like *daf‐2* receptor promotes extracellular aggregate clearance and prolonged ECM maintenance [94, 234]. Mechano‐responsive mediator YAP plays a central role in maintaining ECM homeostasis by sensing and responding to mechanical tension, thereby preserving ECM integrity during ageing and supporting longevity [94]. Together, these findings in *C. elegans* highlight the potential to identify analogous signalling regulators of the exPN in mammals. Furthermore, lifestyle interventions such as exercise may boost extracellular proteostasis capacity, evidenced by elevated plasma clusterin levels following exercise both in mice and humans, which is associated with reduced inflammation [235].

Some reports suggest that upregulating single extracellular PQC factors is sufficient to observe health benefits. Because of its potent antiaggregation and anti‐inflammatory effects, we will focus on strategies identified to boost clusterin's activity as a promising therapeutic approach [236, 237]. In *Drosophila*, heterologous overexpression of human clusterin extended lifespan and improved stress tolerance, in the absence of disease‐related pathologies [238]. Viral‐induced clusterin overexpression in astrocytes ameliorated amyloid pathology and reduced neurotoxicity and gliosis in an AD mouse model [239]. Administration of recombinant clusterin has likewise shown to produce beneficial effects in AD mouse models. Notably, peripheral intravenous delivery of full‐length clusterin reduced amyloid plaque size and neuronal loss despite an absence of detectable clusterin within the brain parenchyma [240]. Intraventricular infusion of a clusterin‐derived peptide reduced Aβ plaque burden, alleviated cerebral amyloid angiopathy and improved memory deficits in an AD mouse model [241]. The peptide, originally selected for its anti‐inflammatory properties [242], resides within an amphipathic helix distant from the clusterin tail motifs responsible for antiaggregation activity, leaving its mechanism of action on Aβ aggregation unclear. The recent identification of a small molecule capable of upregulating clusterin expression is another promising therapeutic avenue. Oral administration of a refined bromodomain and extra‐terminal (BET) inhibitor increased secreted clusterin levels in the hippocampus and improved learning and memory in an AD mouse model, despite no changes in total Aβ levels [85]. Intriguingly, reducing clusterin expression can also confer benefits in AD models, lowering Aβ levels and enhancing behavioural performance [243, 244]. These observations could reflect an activation of alternative extracellular proteostasis mechanisms to compensate for the loss of clusterin. While clusterin is a compelling target for therapeutic development, it also poses potential risks [236, 237]. In particular, its antiapoptotic properties may support tumour progression. Moreover, cell culture experiments reveal that clusterin binds to extracellular tau seeds, which enhances intracellular tau aggregation and toxicity in neurons [245].

Targeting extracellular proteases is another potential strategy to alleviate extracellular protein aggregation and improve extracellular proteostasis. However, the broad substrate specificity and context‐dependent risks associated with these enzymes impose important limitations on their therapeutic use. Several pharmacological and gene therapy approaches have been shown to increase neprilysin activity [246, 247]. Because neprilysin is ineffective at cleaving mature amyloid plaques, approaches that boost its activity are likely to be most beneficial early in disease progression, when reducing monomeric and oligomeric Aβ species should effectively limit aggregate formation. By contrast, MMPs such as MMP9 and MMP2 can degrade amyloid plaques, but their activation in AD poses significant risks, including excessive ECM degradation and blood–brain barrier disruption [248]. Ultimately, successful therapeutic approaches will depend on selectively harnessing protease activity against pathogenic aggregates within defined tissues, avoiding the risks associated with widespread protease upregulation.

Rebalancing ECM remodelling in a targeted manner during ageing is another important strategy to promote healthy ageing, as efficient and regulated matrix turnover is necessary for the clearance of damaged extracellular proteins [8]. However, excessive MMP activity is harmful without parallel collagen synthesis to restore the fragmented ECM, particularly in tissues like the skin, where core ECM components diminish with age. Restoring the ratio of MMP and MMP inhibitor levels with age may be a powerful strategy to support productive ECM remodelling. Increasing levels of the MMP inhibitor, TIMP2, which decline with age, have emerged as a particularly attractive therapeutic approach. Although TIMP2 broadly inhibits MMPs, it is also required for MMP2 maturation in specific contexts. In the hippocampus, TIMP2 deletion impairs MMP2 proteolytic activity causing accumulation of aggrecan puncta [249], whereas systemic delivery of TIMP2 to aged mice improves neuronal function and cognition [250]. Moreover, increased ECM stiffness with age disrupts oligodendrocyte progenitor cell function, and restoring stiffness to youthful levels in mice is sufficient to reverse these deficits [251]. Consistent with these findings, a recent preprint reports that higher plasma TIMP2 levels are associated with better global cognition and larger brain volumes across human cohorts [252]. Importantly, ECM‐remodelling strategies are likely to be most effective combined with treatments aimed at removing AGE damage which impedes remodelling [18]. Several small molecules can break AGE crosslinks, but the first major candidate, alagebrium (ALT711), which targets alpha‐diketone linkages, showed some preclinical efficacy, yet ultimately failed in clinical testing [253, 254]. Current efforts focus on targeting glucosepane, which is more prevalent in humans than alpha‐diketone links [255, 256]. Modulating levels of the ECM glycosaminoglycan, hyaluronan and the hyaluronidase TMEM2 is another option to consider for therapeutic development [257, 258, 259]. Considering the complexity of studying ECM homeostasis in mammals, *in silico* drug selection and *in vivo* screening in *C. elegans* provides a powerful approach to identify novel compounds modulating ECM homeostasis and longevity [260].

Because ECs and proteases act synergistically to preserve the integrity of the extracellular proteome, a coordinated modulation of the exPN is likely to offer the most effective strategy for sustaining extracellular proteostasis and preventing the build‐up of protein damage. In disorders characterised by extracellular aggregation, enhancing extracellular proteostasis may work synergistically with antibody‐based approaches to clear pathological deposits [261]. Importantly, extracellular PQC depends on cellular uptake and lysosomal degradation, pathways that decline with age and are further impaired in protein‐aggregation disorders [262]. Thus, the greatest therapeutic benefit will come from strategies that combine extracellular proteostasis enhancement with reinforcement of lysosomal function. Overall, evidence from multiple model organisms underscores the broad organismal benefits of boosting extracellular proteostasis with age, including improved stress resilience, reduced inflammageing and longevity.

## Conflict of interest

CYE declares to be a co‐founder and shareholder of Avea Life AG and Lichi3 GmbH and is employed by Novartis.

## Author contributions

SRV, IG, BFO, YHC, JRK, CYE and DCD contributed to the conceptualisation, review and editing. SRV, IG, CYE and DCD contributed to the writing—original draft. SRV, BFO, CYE and DCD contributed to the revised version. SRV, JRK and DCD prepared the figures. SI and SRV performed the data analysis.
